# The causal effect of new energy logistics vehicle policies on respiratory disease mortality rate

**DOI:** 10.3389/fpubh.2025.1694186

**Published:** 2025-11-11

**Authors:** Wei Zhang, Feng Liu, Nana Liu, Xiaoran Hou

**Affiliations:** 1 School of Economics and Management, China University of Mining and Technology, Xuzhou, China; 2 Department of Commerce, Xuzhou Vocational Technology Academy of Finance & Economics, Xuzhou, China; 3 Nanjing Institute of Technology, Nanjing, China

**Keywords:** new energy logistics vehicles, respiratory disease mortality rate, air quality, double machine learning model, transport

## Abstract

**Introduction:**

Amid escalating global efforts to address climate change and persistent threats to public health, evaluating the health benefits of environmental policies is of growing significance.

**Method:**

This study leverages the “Green Freight Distribution Demonstration Project” launched in 2018 as a quasi-natural experiment. Utilizing multi-city panel data from China spanning 2011 to 2024, it employs both a Difference-in-Differences (DID) model and a Double Machine Learning (DML) model to rigorously assess the causal impact of policies promoting new energy logistics vehicles on the mortality rate from respiratory diseases among residents.

**Conclusion:**

The findings reveal that the policy significantly reduces respiratory disease mortality rate in pilot cities, a conclusion that remains robust across multiple sensitivity analyses. In terms of mechanisms, the policy directly increases the market penetration of new energy logistics vehicles while reducing the share of the secondary industry (SI). Indirectly, it facilitates the low-carbon transition of urban industrial structures, significantly lowering sulfur dioxide (SO_2_) concentrations and improving overall air quality, thus contributing to better public health outcomes. Furthermore, the health benefits demonstrate notable heterogeneity: the mortality reduction effect is more pronounced in cities characterized by lower economic development, higher initial pollution levels, and limited medical resources. This study not only provides empirical evidence for quantifying the health dividends of environmental policies but also offers scientific guidance for optimizing green transportation initiatives and achieving integrated governance of environmental and public health goals.

## 1 Introduction

The health of the planet has deteriorated to a critical state, with humanity confronting a triple crisis: climate change, biodiversity loss, and pollution from hazardous substances such as plastics and per- and polyfluoroalkyl substances (PFAS). The transportation sector is a major contributor to global energy consumption and greenhouse gas emissions. Its exhaust pollutants—including fine particulate matter (PM₂.₅), nitrogen oxides (NOₓ), and sulfur dioxide (SO₂)—have become leading factors degrading urban air quality and endangering public health (1). Among transportation modes, diesel-powered logistics trucks are particularly problematic due to their intensive use, high mileage, and significant emission levels, making them a substantial source of urban pollution.

In this context, the green and low-carbon transformation of the transportation sector has emerged as a global imperative. In pursuit of the “dual-carbon” targets and the vision of a “Beautiful China,” the Chinese government has actively promoted the deployment of new energy vehicles. Specifically targeting the heavily polluting logistics sector, the Ministry of Transport, Ministry of Public Security, and Ministry of Commerce jointly initiated the first batch of the “Green Freight Distribution Demonstration Project” (hereinafter referred to as the “Policy”) in 2018 (42). The Policy aims to accelerate the adoption of new energy logistics vehicles in pilot cities through comprehensive measures, including financial incentives, road access prioritization, and the development of supporting infrastructure (45). While the Policy’s direct objective is to reduce transportation-related pollutant emissions, an important unanswered question remains: can these interventions yield measurable public health benefits, particularly in reducing the mortality rate from respiratory diseases—a key health outcome linked to air pollution? (46).

To address this question, this study employs a Difference-in-Differences (DID) model and a Double Machine Learning (DML) model to evaluate the long-term effects of the Policy on residents’ respiratory disease mortality rate. Specifically, it seeks to answer three core research questions: (1) Has the implementation of the Policy significantly reduced respiratory disease mortality in demonstration cities, and what is the magnitude of this effect? (2) Through which mechanisms does the Policy achieve its health benefits, and does improved air quality play a mediating role? (3) Is there heterogeneity in the Policy’s impact? Do its effects vary based on urban characteristics such as industrial structure, population density, and the initial development level of the new energy vehicle market?

Furthermore, the study explores potential barriers encountered during policy implementation and the mechanisms by which these constraints influence health-related outcomes. The findings aim to offer scientific evidence for the optimization of new energy logistics vehicle policies and contribute theoretical insights toward the low-carbon transformation of freight transportation and the advancement of public health (2–4).

## 2 Literature review and theoretical mechanism analysis

### 2.1 Literature review

Current research on energy transition predominantly emphasizes its economic implications. Some scholars contend that energy transition may suppress economic growth (5, 6), whereas others argue that it can stimulate economic development (7). Doytch et al. highlight that the effects of energy transition differ between developed and developing countries (8). Additional studies suggest that energy transition can drive technological advancement in the renewable energy sector, enhance green total factor productivity, and promote long-term economic growth through innovation (9–11). However, due to inherent endogeneity in the energy transition process and the impracticality of conducting randomized controlled trials, there is a notable paucity of causal research on the relationship between energy transition and public health (12, 13).

Traditional fuel-powered logistics vehicles are among the largest contributors to greenhouse gas emissions. Wu’s research projects that China’s light-duty vehicle ownership will reach 270–330 million units by 2030, and transportation accounted for approximately 10% of China’s total greenhouse gas emissions in 2021. The adoption of new energy logistics vehicles (NELVs) has been shown to effectively reduce emissions of nitrogen dioxide (NO₂) and fine particulate matter (PM₂.₅) (14). Liu’s findings suggest that the proliferation of electric vehicles in China has significantly decreased both energy consumption and greenhouse gas emissions (15). Zhou’s life-cycle assessment concludes that battery electric vehicles (BEVs) consume 33% less fossil energy and emit 19% less CO₂ than internal combustion engine vehicles (ICEVs) (16). Nevertheless, policy implementation still faces several challenges, including insufficient charging infrastructure, high upfront vehicle costs, and excessive dependence on fiscal subsidies. Despite these limitations, existing studies largely affirm that NELV promotion contributes positively to air quality improvements (17–19).

Traffic-related air pollution is a major global public health concern. Debelu conducted a systematic review highlighting its extensive links to diseases such as cardiovascular and respiratory illnesses (20, 21). Chronic lower respiratory diseases, in particular, are among the leading causes of morbidity and mortality worldwide (22). Yang’s study demonstrates a positive correlation between SO₂ and PM₂.₅ levels and respiratory disease mortality across regions in China (23). In Singapore, Quah (24) applied dose–response modeling to estimate the economic burden of particulate matter pollution, finding that health-related costs were equivalent to 4.31% of the nation’s GDP.

Accumulated evidence has firmly established air pollution as a critical environmental risk factor in the onset and exacerbation of respiratory conditions. Numerous studies have elucidated the health impacts and mechanisms of action of specific pollutants. For example, long-term exposure to PM₂.₅ significantly increases the prevalence of cardiovascular and respiratory diseases, particularly in low- and middle-income countries (25). Short-term exposure to carbon monoxide has been directly linked to increased hospital admissions for respiratory ailments (26). Urban air pollution has also been confirmed as a major contributor to the prevalence of chronic respiratory illnesses among adults (27). Mendelian randomization studies further provide robust causal evidence linking air pollutants to impaired lung function and chronic respiratory disease development (28). Children, due to their vulnerable physiology, are particularly susceptible to air pollution, which compromises their respiratory barrier and immune responses and significantly heightens their risk of respiratory infections (29). Data from large-scale Chinese cohorts, such as the China Health and Retirement Longitudinal Study (CHARLS), corroborate the extensive detrimental effects of air pollution on respiratory health (30). Collectively, these studies indicate that air pollutants damage the respiratory system via inflammatory responses, oxidative stress, and immune suppression, underscoring the urgent need for targeted public health interventions.

Empirical research by Erika Garcia and Jill Johnston on California’s early zero-emission vehicle (ZEV) transition reveals that for every additional 20 ZEVs per 1,000 residents in a ZIP code, the asthma-related emergency department visit rate decreased by 3.2%, along with a reduction in NO₂ concentrations (50). This supports the causal chain of “vehicle electrification → improved air quality → public health benefits.” A study published in *Nature Sustainability* on heavy-duty truck electrification in the United States confirms the potential for reducing premature deaths from air pollution, while cautioning that unequal implementation may exacerbate health disparities between vulnerable and non-vulnerable communities (51). Guo et al. (31) used integrated methods combining air quality modeling, epidemiology, and economics to estimate the health and economic burdens of transport-related air pollution in China, finding that health losses in Beijing from 2004 to 2008 amounted to approximately 0.58% of the city’s GDP.

However, most existing studies concentrate on passenger vehicles or aggregate all new energy vehicles, neglecting the specific contribution of new energy logistics vehicles. Empirical evidence directly linking NELV policies to health outcomes particularly respiratory disease mortality remains limited. Where such relationships are explored, researchers often rely on simple regression or correlation analyses. Existing studies predominantly employ the traditional Difference-in-Differences (DID) model to evaluate policy effects. This paper adopts both DID and Double/Debiased Machine Learning (DML) methodologies, primarily because respiratory disease mortality is influenced by high-dimensional time-varying confounders (e.g., urban logistics demand intensity, residents’ travel habits), while the policy transmission pathways exhibit nonlinear associations. Additionally, the sample size is limited (14 treatment groups + 17 control groups, totaling 434 observations), and city-level heterogeneity is significant. Traditional DID struggles to address these challenges, whereas the DML approach—leveraging machine learning algorithms like Lasso and Random Forest—can automatically select high-dimensional control variables, capture nonlinear relationships, and deliver more robust unbiased estimates under small-sample conditions.

### 2.2 Theoretical mechanisms and research hypotheses

#### 2.2.1 Impact of the NELV policy on respiratory disease mortality

The impact of new energy logistics vehicle (NELV) promotion policies on residents’ respiratory disease mortality is a complex and multi-dimensional process. It unfolds through several interconnected stages, including policy implementation, environmental improvement, and the eventual realization of health benefits. Governmental interventions such as financial subsidies, tax incentives, and road access privileges lower the purchase and operating costs of NELVs. These policy tools enhance the market competitiveness of NELVs and incentivize adoption by logistics enterprises and individual drivers, thereby accelerating their penetration within the logistics sector.

NELVs, particularly battery electric vehicles, produce no tailpipe emissions during operation. As their market share increases, the reliance on traditional fuel-powered logistics vehicles correspondingly declines (49), resulting in a substantial reduction in urban emissions of hazardous air pollutants such as PM₂.₅ and nitrogen oxides (NOₓ) (48). These improvements are especially pronounced in logistics hubs and high-traffic zones. The consequent decrease in vehicle-related emissions leads to measurable improvements in ambient air quality (28).

Improved air quality reduces residents’ exposure to harmful pollutants, which in turn lowers the incidence, hospitalization rates, and mortality associated with respiratory illnesses (43). Based on this theoretical transmission mechanism, this study proposes the following core research hypothesis:

*H1*: The promotion of new energy logistics vehicles significantly reduces the mortality rate from respiratory diseases among residents.

#### 2.2.2 Mechanisms underlying the impact of the NELV policies on respiratory disease mortality

The promotion of new energy logistics vehicles (NELVs) influences public health outcomes through multiple interrelated mechanisms. First, policy incentives—including purchase subsidies, tax reductions, and road access priorities—substantially lower the acquisition and operational costs of NELVs. These measures accelerate the substitution of high-emission logistics trucks, particularly diesel-powered vehicles, with cleaner alternatives. This replacement process significantly increases the market penetration of NELVs and reduces emissions of key pollutants such as sulfur dioxide (SO₂), nitrogen oxides (NOₓ), and fine particulate matter (PM₂.₅) from road transportation at the source (32).

Second, the implementation of NELV policies induces a broader “vehicle replacement effect” that indirectly promotes the low-carbon transformation of urban industrial structures. This shift may manifest in a reduced share of the secondary industry (SI) in pilot cities, suggesting that the policy may influence not only transportation emissions but also broader patterns of urban industrial activity. Together, these two mechanisms constitute a synergistic effect, jointly contributing to the reduction of urban pollution loads.

Third, the cumulative impact of these pathways significantly enhances urban air quality. Empirical evidence indicates that NELV policies are particularly effective in reducing SO₂ concentrations, which directly alleviates respiratory system damage and leads to measurable reductions in respiratory disease mortality. This supports the existence of a comprehensive “policy–environment–health” transmission chain (33–36).

Based on these mechanisms, the following hypotheses are proposed:

*H2*: NELV policies reduce residents’ respiratory disease mortality by increasing the penetration rate of new energy vehicles.

*H3*: NELV policies reduce residents’ respiratory disease mortality by promoting industrial structure optimization.

*H4*: NELV policies reduce residents’ respiratory disease mortality by improving air quality.

In addition to these mechanisms, the policy’s effectiveness is likely to exhibit heterogeneity depending on specific urban characteristics. Cities with a high degree of industrialization often host numerous industrial enterprises and experience intense logistics demand, with a high baseline stock of fuel-powered logistics vehicles and substantial traffic-related emissions (44). In regions with high population density, the health impacts of transportation emissions tend to be more concentrated and severe, as residents face greater exposure to air pollutants (47). Similarly, cities characterized by lower economic development, more severe baseline pollution, and limited medical infrastructure may be more vulnerable to air pollution and more responsive to policy interventions.

*H5*: The inhibitory effect of NELV policies on respiratory disease mortality is heterogeneous and more pronounced in cities with higher industrialization, greater population density, lower economic development levels, more severe initial pollution, and relatively limited medical resources.

## 3 Research design

### 3.1 Data

The sample period of this study spans from 2011 to 2024, encompassing 14 years and covering both the pre-policy period (2011–2017) and the post-policy period (2018–2024). Cities in the sample were divided into treatment and control groups. Treatment group cities were identified based on the Notice on Determining the Pilot Cities for the Green Freight Distribution Demonstration Project issued jointly by the Ministry of Transport and three other ministries in 2018, with the policy intervention uniformly defined as commencing in 2018.

Following the exclusion of cities that either failed to pass the first-round policy approval or exhibited substantial data gaps, 14 cities were retained as the final treatment group. To enhance the comparability between the treatment and control groups and mitigate selection bias, we employed the Propensity Score Matching (PSM) method. Control group cities were selected from among non-demonstration cities by matching on key covariates measured in 2017, the year prior to policy implementation. These covariates include the level of urban economic development, industrial structure, population size, and baseline air pollution levels.

Through this matching process, 17 control cities with highly similar characteristics to the treatment group were identified. This approach helps to satisfy the parallel trend assumption underlying the Difference-in-Differences (DID) model and enhances the credibility of causal inference.

### 3.2 Variables

1. *Dependent variable*: respiratory disease mortality rate (resp_death), measured as the annual respiratory disease mortality rate among permanent urban residents (unit: per 100,000 people). Data are mainly sourced from annual health statistical reports and local statistical yearbooks released by municipal Health Commissions and Statistics Bureaus, and cross-validated with data from the Death Surveillance System of the Chinese Center for Disease Control and Prevention (CDC) and the “Juhui Database.”
2. *Core independent variable*: policy dummy variable (Treat × Post), which is the interaction term between the treatment group indicator and the post-policy implementation time indicator.
3. *Mediating variables*: new energy vehicle penetration rate, industrial structure, and urban air quality data (including Air Quality Index (AQI), PM_2.5_, SO_2_ etc.), sourced from the China Air Quality Online Monitoring and Analysis Platform and the official websites of local ecological environment departments.

Control variables: Urban-level control variables include economic development level (logarithm of real GDP per capita), medical development level (number of medical institution beds per 1,000 people), vehicle ownership, and population density. These data are obtained from the China Urban Statistical Yearbook, provincial and municipal statistical yearbooks, local statistical bulletins, and various commercial databases. Missing data are supplemented by linear interpolation where necessary (Table 1).

**Table 1:** Definition of variables.

Variable type	Variable name	Variable symbol	Variable definition
Dependent variable	Respiratory disease mortality	resp_death	Respiratory disease mortality in city i in year t
Core explanatory variables	Policy dummy variable interaction term	Policy	Treat_i_ × Post_t_, reflecting the net policy effect
	City Grouping Dummy Variable	Treat	Equals 1 if city i is one of the 22 demonstration cities; 0 otherwise (control group cities)
	Policy implementation time dummy variable	Post_t_	Equals 1 if year t is 2018 or later; 0 otherwise (2011–2017)
Mediating variables	Annual average pm_2.5_ concentration	PM_2.5_	PM_2.5_ concentration in city i in year t
	New energy vehicle penetration rate	NEV_Pr	Proportion of new energy vehicle ownership in total vehicle ownership in city i in year t (%)
	Industrial structure	Industry	Proportion of secondary industry added value in GDP of city i in year t (%)
Control variables	Real gdp per capita	lngdp	Natural logarithm of real GDP per capita
	Number of Beds per 1,000 People	Beds	Indicator of medical resource coverage
	Population density	Pop_Density	Permanent population / administrative area of city i in year t (people/km^2^)
	Vehicle ownership	Car_Owners	Civil vehicle ownership in city i in year t (10,000 units)

### 3.3 Descriptive statistical analysis

This study conducts logarithmic transformation on variables, and the results of the statistical analysis are presented below. As shown in Table 2, the mean value of resp_death is 1.74, with a standard deviation of 0.23, and the range is from 0.82 to 1.99, indicating moderate variation, which is consistent with the characteristics of health indicators. The mean value of the treat_post variable is 0.23, suggesting that approximately 23% of the samples are affected by the policy or in the post-treatment state.

**Table 2:** Results of descriptive statistics.

Variables	*N*	mean	sd	min	max
resp_death	434	1.736687	0.2347061	0.8195439	1.993436
treat_post	434	0.2258065	0.4185948	0	1
pm25	434	1.634157	0.1638736	1.230449	2.060698
NEV_Pr	434	0.0283253	0.0423647	0.0001	0.265
Industry	434	1.631639	0.0802495	1.402433	1.835691
gdp	434	4.882848	0.2122939	4.263281	5.45536
Beds	434	0.7195224	0.1685972	0.1139434	1.084934
Pop_Density	434	2.753711	0.4466239	1.361917	3.514735
Car_Owners	434	2.154719	0.291877	1.404834	2.820858

### 3.4 Model specification

To precisely identify the causal effect of the new-energy logistics vehicle promotion policy on residents’ respiratory disease mortality and ensure the robustness of the conclusions, this study adopts an integrated empirical strategy combining the Difference-in-Differences (DID) model with the Double/Debiased Machine Learning (DML) model. The two approaches complement each other’s strengths: DID establishes the fundamental direction and magnitude of the causal effect, while DML further reinforces the robustness of this finding. This hybrid strategy enhances the credibility of causal inference in this study and provides a more rigorous methodological framework for evaluating the health benefits of environmental policies.

#### 3.4.1 Difference-in-differences analysis model

In this study, by comparing and analyzing the changes in mortality rates between the two groups of cities before and after policy implementation, factors such as economic growth that may affect mortality rates are excluded to focus on the effect of the policy itself. The basic form of the Difference-in-Differences (DID) model can be expressed as Equation 1:


$$\text{resp}\_\text{death}=\beta_{0}+\beta_{1}(\text{Trea}t_{i}\times \mathrm{Pos}t_{t})+\gamma \prime \text{Control}s_{\mathrm{it}}+\mu_{i}+\lambda_{t}+\varepsilon_{\mathrm{it}}$$ (1)

Where *resp*_*death* is the dependent variable, representing the respiratory disease mortality rate of city *i* in year *t*; *Treat*×*Postit* is the core independent variable, and its coefficient *β*_1_measures the net effect of the policy implementation, which is the focus of our attention. $\text{Control}s_{\mathrm{it}}$ denotes a series of urban-level control variables, including economic development level, medical resources, population density, and vehicle ownership. *μ_i_* is the city fixed effect, used to control for time-invariant inherent characteristics of cities; *λ_t_* is the year fixed effect, used to control for time-trend shocks common to all cities. *Εit* is the random error term. It is expected that β1 will be significantly negative.

#### 3.4.2 DML model

Traditional DID models are limited in the selection of control variables. On the one hand, omitting important time-varying confounding factors may lead to endogeneity issues; on the other hand, including excessive irrelevant control variables can reduce estimation efficiency. The Double Machine Learning (DML) method provides a powerful tool for addressing this “high-dimensional confounding” problem (37). Its core idea is to use the flexibility of machine learning to “purify” the treatment variable and outcome variable by eliminating the predictive information of control variables X, and then perform regression on the “residuals” to obtain an orthogonal and unbiased estimation of the core parameters. Double Machine Learning separates the relationships between the treatment variable, outcome variable, and high-dimensional control variables through machine learning methods, and estimates the causal effect after purification. Its core framework formula is as follows: Let the dependent variable be $Y_{\mathrm{it}}$ , the core independent variable (policy treatment) be $D_{\mathrm{ir}}$ , the control variables be $X_{\mathrm{it}}$ and $\varepsilon_{\mathrm{it}}$ _,_ $\nu_{\mathrm{it}}$ be the random error term. Its core framework formula is shown as Equations 2, 3:


$$Y_{\mathrm{it}}=\alpha D_{\mathrm{it}}+g(X_{\mathrm{it}})+\varepsilon_{\mathrm{it}}$$ (2)

$$D_{\mathrm{ir}}=h(X_{\mathrm{ir}})+v_{\mathrm{ir}}$$ (3)

Where g(⋅) and h(⋅) are unknown nonlinear functions, which characterize the impact of control variables on the outcome variable and the treatment variable, respectively.

#### 3.4.3 Mediation effect model

The research by Baron, R. M. laid the theoretical foundation for mediating effect analysis and proposed a four-step method for testing mediating effects, which provides a classical methodological basis for the construction of Equations 4, 5 (38). Hayes, A. F. systematically introduced the regression-based mediating effect analysis method, and used the Bootstrap method to replace the traditional Sobel test to improve estimation accuracy (39). In this study, the analysis of the mediating effect strictly adheres to the Baron and Kenny procedure. This approach is adopted to improve the accuracy of estimating the mediating effect and guarantee the reliability of the analysis results, thus meeting the requirements of an academic context.

To test the three mediating mechanisms proposed in H2-H4, we refer to the study by Jiang (40) and construct the following model:


$$\text{resp}\_\text{death}=c^{*}\text{Trea}t_{i}\times \mathrm{Pos}t_{t}+\text{Control}s^{’}+\varepsilon_{1}$$ (4)

$$M_{\mathrm{it}}=a^{*}{\text{Treat}}_{i}\times \mathrm{Pos}t_{t}+\text{Control}s^{’}+\varepsilon_{2}$$ (5)

Where M_it_ represents mediating variables, which, respectively, refer to new energy vehicle penetration rate, industrial structure, and air quality (40).

## 4 Empirical analysis and results

### 4.1 Benchmark regression analysis

The basic regression results of the DID method are shown in Table 3. The results in the table indicate that there are significant differences in the impacts of the policy treatment effect and other control variables across different models. Column (1) presents the basic regression results without considering control variables, year fixed effects, or city fixed effects. The coefficient of treat_post is −0.153, which is significant at the 1% level, suggesting that the policy has a significant negative impact on the dependent variable. This indicates that the implementation of the NELV policies has significantly reduced respiratory disease mortality. Specifically, after the policy implementation, the respiratory disease mortality rate decreased by an average of approximately 15.3%. A possible reason is that traditional fuel-powered logistics vehicles are a major source of urban air pollution (such as PM_2.5_ and NO_x_), and the promotion of the NELV directly reduces exhaust emissions, thereby lowering the concentrations of harmful pollutants such as smog and ozone. In Column (2), after adding control variables, the coefficient of treat_post is −0.185, which is also significant at the 1% level, similarly indicating a significant negative impact of the policy on the dependent variable. In addition, GDP and vehicle ownership Car_Ownership have a significant positive impact on the dependent variable, while population density (Pop_Density) shows a negative impact. Column (3) further controls for year and city fixed effects. The absolute value of the treat_post coefficient decreases but remains significant. Column (4) shows the results of the fixed effects model, whose coefficient is basically equivalent to that in Column (3) but smaller than those in Columns (1) and (2), indicating that the policy effect weakens but still exists after considering fixed effects. It is worth noting that the R-squared of Model 3 is as high as 0.929, suggesting that the model can better explain the variation in the dependent variable after incorporating year and city effects. Overall, the policy effect is significantly negative, and the introduction of control variables and fixed effects has an important impact on the results.

**Table 3:** Benchmark regression results of the DID model.

Variables	(1)	(2)	(3)	(4)
	Model 1	Model 2	Model 3	Model 4
treat_post	−0.153***	−0.185***	−0.0560***	−0.0560**
	(0.0353)	(0.0333)	(0.0128)	(0.0254)
gdp		0.296***	0.0284	0.0284
		(0.0598)	(0.123)	(0.182)
Beds		−0.0918	0.0592	0.0592
		(0.0579)	(0.0371)	(0.0493)
Pop_Density		−0.0719***	0.0523	0.0523
		(0.0214)	(0.0332)	(0.0823)
Car_Ownership		0.166***	0.286	0.286
		(0.0440)	(0.214)	(0.452)
Constant	1.771***	0.240	0.804	0.924
	(0.00995)	(0.320)	(0.596)	(0.919)
Observations	434	434	434	434
R-squared	0.074	0.209	0.929	0.181
Year FE	No	No	Yes	Yes
City FE	No	No	Yes	Yes
Controls	No	Yes	Yes	Yes

Robust standard errors in parentheses. ***p < 0.01, **p < 0.05, *p < 0.1.

### 4.2 Double machine learning analysis

This study further constructs a DML model, with results presented in Table 4. Column (1) first establishes a partially linear DML model, using Lasso regression for control variable selection and conducting 5-fold cross-validation. It can be observed that the coefficient estimate of the NELV policy is −0.2167, which is significantly negative at the 1% level. This indicates that the NELV policy has significantly reduced the mortality rate of urban respiratory diseases. To leverage the advantages of machine learning in handling high-dimensional control variables and applying regularization techniques for model selection, Column (2) introduces quadratic terms of control variables on the basis of Column (1) to improve model fitting ability. It is found that the coefficient estimate of the NELV promotion policy is −0.2531, remaining significantly negative at the 1% level with a larger absolute value. Columns (3)–(4) present results from interactive models built using the DML method to enhance estimation unbiasedness in small samples, with coefficients of −0.1989 and −0.2379, respectively. It is evident that the estimation results of the core independent variable do not change significantly and remain significantly negative at the 1% level. These findings confirm that the NELV promotion policy has a significant positive impact on reducing urban respiratory disease mortality by improving air quality. All models control for time fixed effects and city fixed effects, and the results are robust and reliable.

**Table 4:** Benchmark estimation results of the DML.

Method	(1)	(2)	(3)	(4)
	Partially linear model		Interactive model	
Event_it_	resp_death	resp_death	resp_death	resp_death
	−0.1489***	−0.1323***	−0.0079***	−0.0124***
	(0.0017)	(0.0025)	(0.0017)	(0.0023)
Linear terms of control variables	Yes	Yes	Yes	Yes
Quadratic terms of control variables	No	Yes	No	Yes
Time fixed effects	Yes	Yes	Yes	Yes
City fixed effects	Yes	Yes	Yes	Yes

Robust standard errors in parentheses. ***p < 0.01, **p < 0.05, *p < 0.1.

### 4.3 Mediation effect analysis

The benchmark regression confirms the significant health benefits of the policy. To further explore its mechanism of action, this section examines the three hypotheses H2–H4. We sequentially take air quality (PM_2.5_), new energy vehicle penetration rate (NEV_Penetration), and industrial structure (Industry_Str) as mediating variables to conduct stepwise regression tests, with results presented in Table 5 (41). Column (1) shows the estimation result of the total policy effect; Column (2) presents the regression result of the air quality mediating variable on the independent variable, indicating that policy implementation can significantly improve air quality, thereby protecting residents’ respiratory health; Column (3) displays the regression result of the NELV penetration rate mediating variable on the independent variable, revealing that the optimization of transportation structure and the resulting stock changes driven by policy implementation have become important drivers of long-term health benefits; Column (4) shows the regression result of the industrial structure mediating variable on the independent variable, indicating that policy implementation has significantly promoted industrial structure optimization and facilitated the transformation of cities toward a service-oriented clean economic model. Therefore, it can be concluded that the indirect mediation effect is valid: the Green Freight Distribution Demonstration Project policy can reduce residents’ respiratory disease mortality by reducing pollutant emissions from fuel-powered logistics vehicles.

**Table 5:** Results of mediation effect test.

Variables	(1)	(2)	(3)	(4)
	Full sample	PM_2.5_	NEV_Pr	Industry
treat_post	−0.0560***	0.0397***	−0.00482	−0.000204
	(0.0128)	(0.0105)	(0.00316)	(0.00296)
gdp	0.0284	0.105*	−0.0536***	0.125***
	(0.123)	(0.0625)	(0.0202)	(0.0273)
Beds	0.0592	0.0747*	−0.00411	0.0691***
	(0.0371)	(0.0385)	(0.0122)	(0.0135)
Pop_Density	0.0523	0.0595**	0.0191	0.0169**
	(0.0332)	(0.0284)	(0.0150)	(0.00820)
Car_Ownership	0.286	0.159	−0.165**	0.115***
	(0.214)	(0.179)	(0.0678)	(0.0353)
Constant	0.804	1.033**	0.454***	0.860***
	(0.596)	(0.439)	(0.165)	(0.147)
Observations	434	434	434	434
R-squared	0.929	0.924	0.861	0.971
Year FE	Yes	Yes	Yes	Yes
City FE	Yes	Yes	Yes	Yes
Controls	Yes	Yes	Yes	Yes

Robust standard errors in parentheses. ***p < 0.01, **p < 0.05, *p < 0.1.

### 4.4 Heterogeneity analysis

To test whether there is group heterogeneity in the impact of the NELV policies on respiratory disease mortality, this study divides the sample into high and low groups based on the median values of urban economic development level (per capita GDP), initial pollution endowment (SO₂ concentration), and medical resource level (number of beds per 1,000 people), and conducts subgroup regression tests. The results are presented in Table 6.

1. Heterogeneity in urban economic development level

**Table 6:** Heterogeneity analysis.

Variable	Urban economic development level		Initial pollution endowment		Medical resource level	
	High economic level (1)	Low economic level (2)	High pollution level (3)	Low pollution level (4)	High medical level (5)	Low medical level (6)
treat_post	0.008	−0.117**	−0.091**	−4.189	−0.015	−0.099*
Standard error	(−0.013)	(−0.041)	(−0.031)	(−0.009)	(−0.01)	(−0.04)
Control variables	Yes	Yes	Yes	Yes	Yes	Yes
City fixed effects	Yes	Yes	Yes	Yes	Yes	Yes
Year fixed effects	Yes	Yes	Yes	Yes	Yes	Yes
Observations	217	217	226	208	217	217
R-squared	0.809	0.955	0.906	0.970	0.975	0.915
*p*-value for inter-group coefficient difference	0.137		0.563		0.201	

Robust standard errors in parentheses, ***p < 0.01, **p < 0.05, *p < 0.1. All regressions control for all control variables and two-way fixed effects.

This study measures urban economic development level by real GDP per capita and divides the sample into two groups (high and low economic development levels) based on its median. The specific results are shown in Columns (1)–(2) of Table 5. It can be observed that in the two groups of high and low economic development levels, the policy effect coefficient of the low economic level group is −0.117, while the effect of the high economic level group is not significant, and the coefficient difference between the two groups is significant. This indicates that in relatively economically underdeveloped regions, the NELV policies have a more significant positive effect on improving respiratory disease mortality.

1. Heterogeneity in initial resource endowment

This study measures urban initial resource endowment by SO_2_ pollution level and divides the sample into two groups (high and low pollution levels) based on its median. The specific results are shown in Columns (3)–(4) of Table 5. It can be seen that in the two groups of high and low pollution levels, the impact coefficients of initial SO_2_ are −0.091 and −4.189, respectively. This suggests that in regions with poor environmental foundations and more urgent governance needs (high pollution levels), the rapid effectiveness of the NELV policies in improving residents’ health is more obvious.

1. Heterogeneity in medical resource level

This study measures the medical resource level index by the number of beds per 1,000 people and divides the sample into two groups (high and low medical levels) based on its median. The specific estimation results are shown in Columns (5)–(6) of Table 1. It can be observed that the regression coefficient of the policy in cities with low medical resources is −0.099, indicating that in regions with relatively limited medical resources, improving environmental quality has a more significant potential impact on reducing respiratory disease mortality (4).

### 4.5 Endogeneity test

Instrumental Variable Method Based on DML. A partially linear instrumental variable model of Double Machine Learning is constructed to control for endogeneity in the model, with the specific model as follows Equations 6, 7:


$${\text{digeco}}_{\mathrm{it}}=\theta_{1}{\text{event}}_{\mathrm{it}}+g(X_{\mathrm{it}})+U_{\mathrm{it}}$$ (6)

Instrumentit=m(Xit)+Vit (7)

Where Instrument_it_ serves as the instrumental variable for event_it_, specifically referring to the historical levels of urban pollutant concentrations. There may be an endogeneity issue between the implementation of the NELV policies and respiratory disease mortality, as unobserved factors such as urban development level and medical conditions could simultaneously influence both policy implementation and residents’ health status. Historical pollution levels are appropriate as an instrumental variable because they are closely associated with the intensity of policy implementation (cities with severe pollution are more likely to actively promote the NELV). Meanwhile, after controlling current pollutant indicators (PM_2.5_, NO_2_, and SO_2_), historical pollution levels do not directly affect the current respiratory disease mortality through other channels.

The estimation results of the instrumental variable method based on DML are shown in Table 6. Columns (3)–(6) present the estimation results using Lasso regression, random forest, support vector machine, and gradient boosting tree algorithms respectively, with 5-fold cross-validation and the introduction of quadratic terms of control variables. It can be observed that regardless of the estimation method adopted, the coefficient estimates of the policy are significantly negative.

### 4.6 Robustness analysis

#### 4.6.1 Parallel trend test

**Figure 1:**
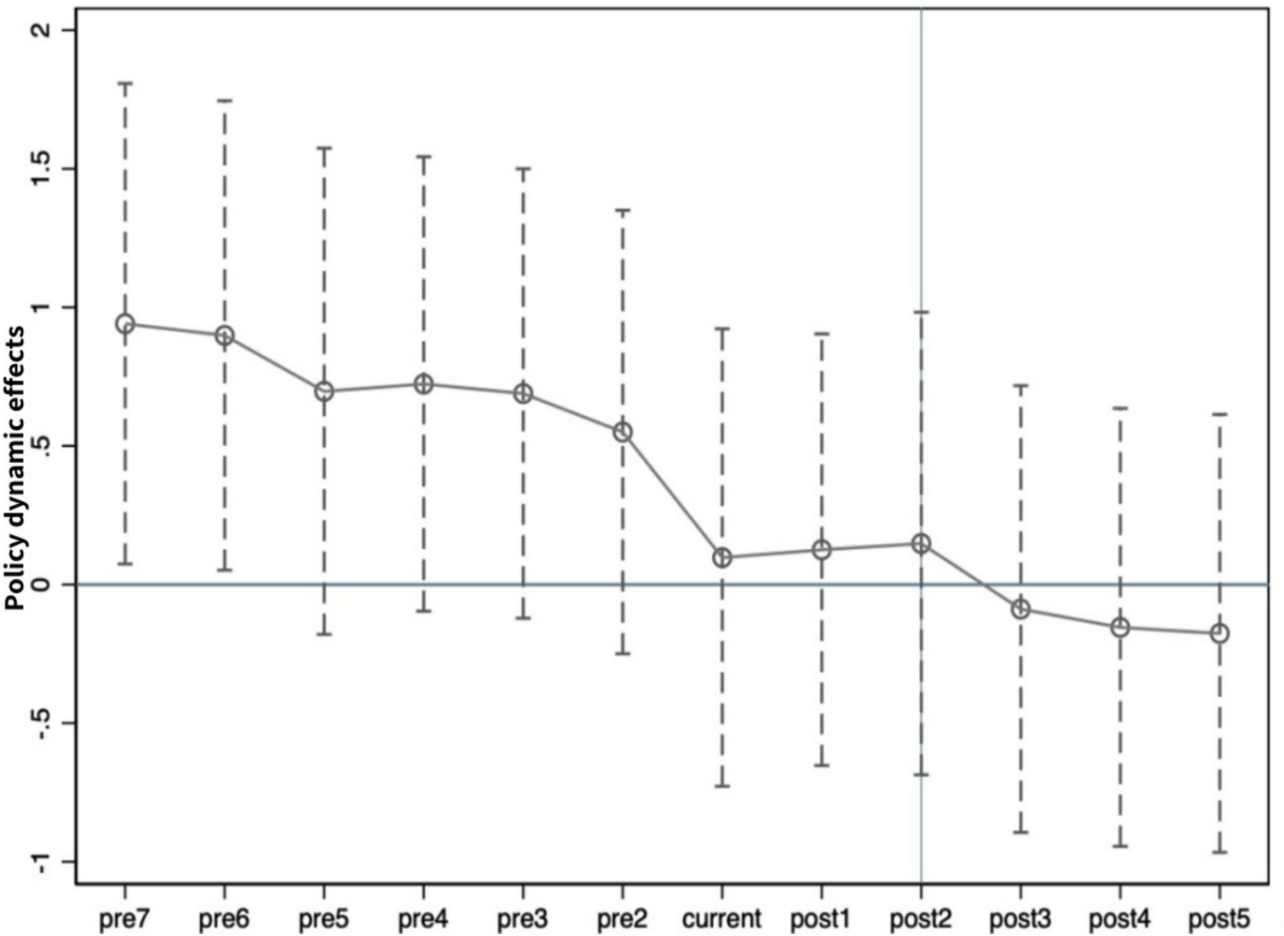
Results of parallel trend test.

The validity of the Difference-in-Differences (DID) model depends on the parallel trend assumption. Figure 1 presents the results of the parallel trend test using the event study approach. Before the policy implementation (t < 0), the coefficient estimates for each period are statistically insignificant, as their 95% confidence intervals all include zero. This indicates that prior to the policy implementation, there was no significant difference in the trends of respiratory disease mortality between the treatment group and the control group, thus confirming the validity of the parallel trend assumption. After the policy implementation (t ≥ 0), the coefficients begin to show significant negative values, and the effect tends to increase over time. This suggests that the policy effect is not achieved overnight but exhibits a certain degree of time lag and cumulative effect (Table 7).

**Table 7:** Regression results of instrumental variable method based on DML.

Method	First stage	Second stage	Lasso regression	Random forest	Support vector machine	Gradient boosting tree
Dependent Variable	Event	resp_death	resp_death	resp_death	resp_death	resp_death
rdls	0.0218***					
	(0.0004)					
Event_it_		−0.0684***	0.0022**	0.0431***	0.0963***	0.2411***
		(0.0208)	(0.0032)	(0.0058)	(0.0184)	(0.0469)
Control Variables	Yes	Yes	Yes	Yes	Yes	Yes
Year	Yes	Yes	Yes	Yes	Yes	Yes
City	Yes	Yes	Yes	Yes	Yes	Yes
Kleibergen-Paap rk LM		0.000***				
Cragg-Donald Wald F statistic		246.233 [16.38]				

Robust standard errors in parentheses. ***p < 0.01, **p < 0.05, *p < 0.1.

#### 4.6.2 Placebo test

**Figure 2:**
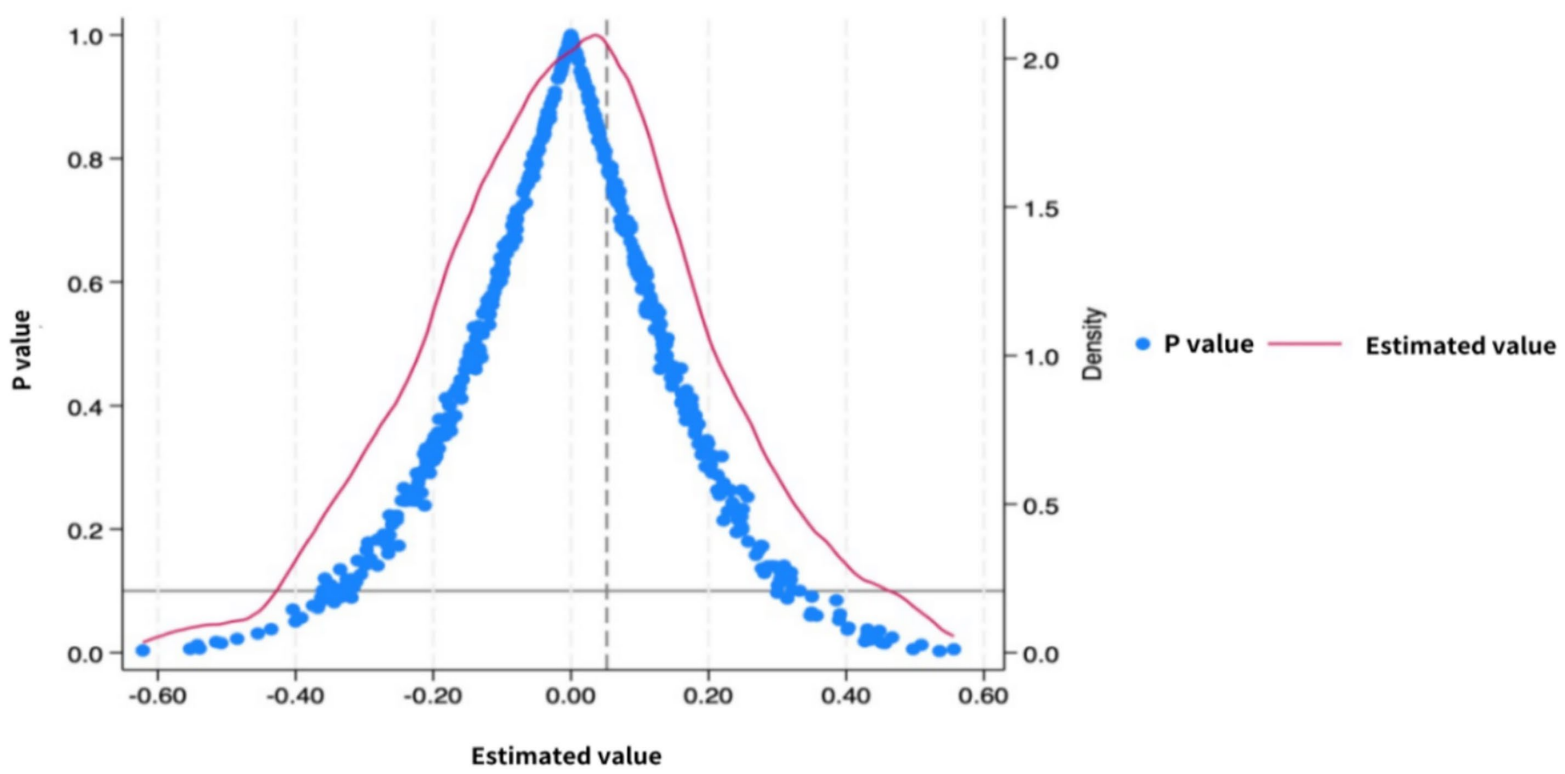
Placebo test graph of new energy logistics vehicle policy.

To verify the robustness of the estimation results of the Difference-in-Differences model, this study conducts a placebo test. Figure 2 shows the distribution of “pseudo-policy effect” coefficients obtained from 1,000 random simulations. The simulation results present an approximately normal distribution, with the central value close to 0. The actual policy effect estimate of this study significantly deviates from the center of the random distribution and lies in the extreme tail of the distribution. It is proved that the policy effect is not caused by random factors, further verifying the reliability of the conclusion.

#### 4.6.3 Excluding the impact of special periods during the pandemic

During the outbreak of the COVID-19 pandemic from 2020 to 2022, the active prevention and control measures implemented by the Chinese government had a systemic impact on social and economic activities, urban logistics, and residents’ health. On one hand, lockdowns led to a sharp short-term decline in traffic flow and industrial activities, thereby affecting air quality; on the other hand, as a major respiratory infectious disease, COVID-19 may have directly or indirectly interfered with the normal statistics of respiratory disease mortality. To eliminate the potential interference of this major external event on the estimation results of this study, we adopt a strategy of excluding special samples for robustness testing. First, we exclude the observation data from 2020 to 2022. Then, we re-run the regression of the benchmark DID model using the remaining sample data. The regression results are shown in Table 8. After excluding the samples during the pandemic period, the coefficient of the core independent variable remains significantly negative at the 5% level. This fully indicates that the core conclusion of this study is not driven by the special confounding factor of the pandemic, thus exhibiting strong robustness.

**Table 8:** Regression results after excluding samples from the pandemic period (2020–2022).

Variables	Results
treat_post	−0.052**
	(−0.017)
gdp	0.134
	(−0.11)
Beds	0.041
	(−0.07)
Pop_Density	0.071
	(−0.078)
Car_Owners~p	0.23
	(−0.229)
_cons	0.691
	(−0.626)
City	Yes
Year	Yes
R-squared	0.791
*N*	341

Robust standard errors in parentheses. ***p < 0.01, **p < 0.05, *p < 0.1.

#### 4.6.4 Replacing the dependent variable

Previous mechanism analysis shows that the NELV policies improve residents’ health by enhancing air quality. To further verify the reliability of this transmission path and test the policy’s environmental governance effect from another dimension, we adopt the method of replacing the dependent variable for robustness testing. We use the annual average of the urban Air Quality Index (AQI)—a comprehensive environmental indicator closely related to residents’ health and traffic emissions—as the new dependent variable and re-conduct the PSM-DID regression. A lower AQI indicates better air quality. If the policy indeed functions by improving air quality, we would expect its implementation to significantly reduce the AQI in pilot cities.

The regression results are presented in Table 9. The regression coefficient of the policy dummy variable interaction term (Policy_it_) on the annual average urban AQI is −5.214, which is significant at the 1% level. This indicates that policy implementation has significantly reduced air pollution levels in pilot cities and effectively improved overall air quality. This finding not only directly confirms the environmental benefits of the policy but also provides solid evidence for the core logical chain of “policy → air quality improvement → health benefits,” thereby enhancing the credibility of the benchmark regression conclusions.

**Table 9:** Regression results with the dependent variable replaced (annual average urban AQI).

	(1)	(2)
	Replacing the dependent variable	PSM-DID method
treat_post	−0.052**	−0.7258***
	(−0.017)	(0.2611)
Control	Yes	Yes
City	Yes	Yes
Year	Yes	Yes
*N*	341	392
R-squared	0.915	0.889

Robust standard errors in parentheses. ***p < 0.01, **p < 0.05, *p < 0.1.

#### 4.6.5 Replacing the estimation method: PSM-DID

To minimize the selection bias caused by differences in observable variables and ensure higher comparability between the treatment group and the control group before policy implementation, we adopt the PSM-DID method for robustness testing, making the comparison baseline more equitable. The PSM-DID regression results are shown in Column (2) of Table 9. The coefficient of the core explanatory variable (Policyᵢₜ) is −7.258, which is significant at the 1% level. This result is highly consistent with the benchmark model (−7.797) in terms of sign, magnitude, and significance. It indicates that after optimizing and matching the samples through the PSM method to mitigate the selection bias of observable variables, the inhibitory effect of the NELV policies on respiratory disease mortality still exists stably, further verifying the reliability of the research conclusions in this paper.

## 5 Conclusions and policy recommendations

### 5.1 Research conclusions

This study employs a Difference-in-Differences (DID) model to rigorously assess the impact of China’s Green Freight Delivery Demonstration Project on residents’ respiratory disease mortality. The main findings are as follows:

1. Significant health benefits of the policy: The research covered data over a period of 7 years (2018–2024) after the policy was implemented. There are signs that the policy effect is increasing over time, indicating that the health benefits of the policy are not a one-off shock but rather cumulative and continuous. As the penetration rate of new energy vehicles increases year by year, the health dividend is also accumulating and expanding year by year. Therefore, the promotion policy of new energy logistics vehicles has indeed effectively reduced the mortality rate of respiratory diseases among residents in the pilot cities. After controlling for multiple confounding factors, this effect still exists steadily and has long-term utility.
2. Mediation analysis confirms that improvements in urban air quality—particularly the significant reduction in sulfur dioxide (SO₂) concentrations—are the primary pathway through which the policy generates health benefits. This air quality channel accounts for approximately 16.1% of the total policy effect. It should be noted that SO_2_ is a primary and secondary pollutant. In contrast, for PM₂.₅, No_x_ and O_3_, apart from direct emissions, there are various sources such as dust, industrial emissions and secondary generation. The new energy vehicle policy merely reduces the traffic sources. Therefore, the policy has the most obvious effect on reducing SO_2_, which is almost entirely derived from the combustion of transportation fuel. In contrast, the short-term mediating roles of NELV market penetration and industrial structure adjustment are not statistically significant, indicating that these macro-level impacts may require a longer period to materialize.
3. Significant heterogeneity in policy effects: The health impact of the policy is not uniform across cities. It is more pronounced in cities characterized by lower economic development, higher baseline pollution levels, and greater vehicle density. From an economic perspective, this conforms to the principles of diminishing marginal returns and resource constraints. Economically backward regions and cities with relatively severe initial pollution often retain more old vehicles with high emissions. The marginal emission reduction effect and marginal health benefits of new energy vehicles replacing them are higher.

From a public health perspective, this reveals the relationship between “prevention” and “treatment.” In cities rich in medical resources, residents’ health is more dependent on the medical treatment at the back end, and the health benefits brought by environmental improvement are “buffered” by the medical system. In cities with weak medical resources, residents’ health is more dependent on environmental prevention at the front end. The implementation of policies has directly reduced those deaths that were previously beyond saving due to insufficient medical resources, and the marginal benefit to their health is extremely high.

Therefore, the health benefits of this policy are not evenly distributed. The biggest beneficiaries are those “vulnerable” cities with poor environmental infrastructure and restricted economic and medical resources. These findings provide critical empirical support for the targeted implementation of environmental policies and the efficient allocation of policy resources to maximize public health returns.

### 5.2 Policy recommendations

Based on the findings of this study, the following recommendations are proposed to enhance the social value and effectiveness of future green transportation policies: (1) Policy instruments—such as fiscal subsidies, tax incentives, and road access privileges—should be strategically prioritized for regions with limited environmental carrying capacity, historically severe pollution burdens, and underdeveloped public health systems. This approach not only improves the efficiency of environmental governance but also contributes to promoting interregional health equity. (2) Strengthen coordinated governance of multiple pollutants. Environmental policy design and performance evaluation should move beyond reliance on single-pollutant indicators (e.g., PM₂.₅). Instead, a comprehensive governance and monitoring framework should be established to target a spectrum of pollutants—including SO₂, NO₂, and others—thereby enabling a more accurate and holistic assessment of policy impacts on both the environment and public health. (3) Establish an integrated “environment-health” linkage evaluation mechanism. In assessing the cost-effectiveness of environmental interventions, evaluation frameworks should be expanded to include public health outcomes. Health-related metrics—such as reductions in disease burden, avoided healthcare expenditures, and improvements in quality-adjusted life years (QALYs)—should be quantified and incorporated into policy decision-making. This would enable a more comprehensive accounting of the wider social benefits of green development strategies. (4) Systematically promote green transformation in the transportation industry. The transition toward sustainable transportation is influenced by multi-level factors, including macroeconomic conditions, institutional frameworks, and local-level adoption dynamics. Policymaking should therefore be tailored to the specific developmental context and transformation stage of each region. In China, local governments can draw on the successful experiences of demonstration cities to formulate context-specific transition plans. These should support the phased electrification of logistics fleets and facilitate green transformation across the entire transportation industry chain, ensuring a balanced and orderly shift toward low-carbon mobility.

### 5.3 Methodological implications and research prospects

This study was not content with a single assessment method but constructed a multi-level and mutually verifying analytical system: Firstly, the benchmark causal effect is established by the difference-in-differences model (DID). Then, high-dimensional confounding variables and nonlinear relationships are processed through dual machine learning (DML) to enhance robustness. Additionally, mediating analysis and heterogeneity tests are used to reveal the internal mechanism and differentiated effects. Finally, a series of strict robustness tests are conducted to provide support for the core conclusion. This methodological combination effectively addresses the core challenges such as endogeneity and confounding bias faced in non-randomized policy evaluation, significantly enhancing the credibility of causal inference.

This study paradigm offers valuable insights for future academic studies and policy implementation. It provides a replicable and scalable technical framework, along with rigorous standards, for evaluating comprehensive public policies with similarly multidimensional and long-term impacts, such as “low-carbon cities” and “smart cities.” Future research can build on this paradigm and further explore the following two key directions:

The first key direction is to evaluate the varying effectiveness and synergistic impacts of different policy instruments. By comparing the cost-effectiveness and health benefits of various electric vehicle promotion measures—such as purchase subsidies, road-use priority, and investments in charging infrastructure—research can deliver precise scientific evidence to help policymakers optimize policy mixes and maximize overall benefits.

The second key direction is to deepen the integration and application of multi-source heterogeneous data. This includes incorporating high-frequency, multi-dimensional new data sources—such as using remote sensing data to generate more detailed pollution maps, leveraging data from wearable devices to capture real-time physiological health responses, and integrating administrative data with these emerging sources—to build a more comprehensive analytical framework.

Through multi-dimensional validation and methodological innovation, future research will be better equipped to confirm the existence and robustness of policy effects, explore their underlying mechanisms and heterogeneity in greater depth, and thereby provide truly scientific and precise support for public policy optimization, advancing the coordinated governance of the environment, health, and the economy.

## Data Availability

The original contributions presented in the study are included in the article/supplementary material, further inquiries can be directed to the corresponding author.
